# OrnAsia: A dataset of asian ornaments for image classification and cultural identification

**DOI:** 10.1016/j.dib.2025.112195

**Published:** 2025-10-18

**Authors:** Md. Darun Nayeem, Saima Zannat Sraboni, Shejuti Shithi Biswas, Md. Masudul Islam

**Affiliations:** Bangladesh University of Business and Technology, Mirpur, Dhaka, 1216, Bangladesh

**Keywords:** Jewellery, Computer vision, Object detection, Deep learning, Fine-grained classification, Cultural heritage, Visual dataset

## Abstract

This article presents a curated dataset comprising 1088 high-resolution original images of six traditional Asian ornaments: Bangles, Rings, Earrings, Tikka, Necklaces, and Payel. The dataset was developed to support the classification and identification of culturally significant jewellery using computer vision and artificial intelligence techniques. All images were captured in real-world settings, including local markets and remote areas of Mirpur, Dhaka, Bangladesh, using smartphone cameras under natural lighting conditions. The collection process ensured variation in lighting angles, perspectives, and ornament positioning to enhance the dataset’s robustness and realism. The data was organized into six distinct categories, with both training and testing subsets clearly defined to facilitate machine learning model development. Each class reflects unique visual features commonly found in ethnic accessories worn across South Asia. The dataset emphasizes diversity in design and background context, making it valuable for research in fine-grained image classification, object detection, and cultural artifact recognition. The dataset serves multiple purposes within the AI field: training deep learning models for ornament recognition, evaluating performance across different neural network architectures, and enabling the development of real-time identification systems. Its availability on a public platform (Mendeley Data) supports open research, reproducibility, and global collaboration. The structured image set can be directly reused for benchmarking classification algorithms, developing lightweight AI models suitable for mobile applications, and enriching visual datasets in cultural heritage research. It also fills a noticeable gap in ornament-related datasets, which are typically limited in scope, cultural context, and image diversity. By offering a balanced mix of traditional ornament types, verified labelling, and application-ready formats, this dataset contributes meaningfully to the advancement of AI applications in fashion technology, cultural preservation, and intelligent retail systems.

Specifications Table

**Table:** 

Subject	Computer Sciences
Specific subject area	Computer vision, image processing, and deep learning for cultural object classification and ornament recognition tasks.
Type of data	Raw, Image
Data collection	Images were collected using Realme Narzo 20 Pro and Realme Narzo 50 smartphones from local markets and rural areas in Mirpur, Dhaka, Bangladesh. Ornaments were photographed under natural lighting between 25 °C–28 °C from multiple angles to capture diverse visual features. Data were manually sorted into six categories. Low-quality, blurry, or duplicate images were excluded. All images were resized and normalized for consistency in model training and testing.
Data source location	Latitude 23.8103° N, Longitude 90.4125° E
Data accessibility	Repository name: **Mendeley Data** Data identification number: 10.17632/sfrd2bzdgb.2 Direct URL to data: https://data.mendeley.com/datasets/sfrd2bzdgb/ To access the dataset, visit the provided Mendeley Data preview link. On that page, you will find the “Download” button. Accept the terms if prompted, and download the ZIP archive. The archive contains 1088 JPG raw files sorted into six class folders. These files can be directly imported into machine‑learning frameworks. No request for additional access is required securely.
Related research article	None

## 1 Value of the Data

• This dataset addresses the lack of representation of Asian cultural artifacts in existing computer vision datasets. By focusing on six traditional ornament types—Bangles, Earrings, Necklaces, Payels, Rings, and Tikkas—it contributes to developing more inclusive and culturally aware AI systems.
• Researchers and developers can reuse this dataset to build automated tools for cataloging, archiving, and authenticating traditional jewelry. It also supports use in virtual museum exhibitions, heritage documentation, and interactive educational platforms.
• The dataset provides a valuable benchmark for evaluating the robustness and accuracy of state-of-the-art computer vision architectures—including CNNs and Vision Transformers—on culturally specific image data.
• Bridging computer science, anthropology, and design, this dataset encourages research in visual culture, ornament symbolism, and historical design analysis. It supports novel applications like generative design, cultural pattern recognition, and style transfer in digital art.
• The diversity of angles, backgrounds, and lighting in the images simulates real-world variability, making the dataset suitable for training models used in e-commerce platforms, AR try-on tools, and mobile applications for ethnic fashion retail.

## 2 Background

Ornaments have long held cultural, aesthetic, and symbolic significance across civilizations [1], with Asian ornaments showcasing some of the world’s most intricate and diverse craftsmanship [2]. These items, ranging from Bangles and Earrings to Necklaces and Tikkas, are worn for ceremonial, religious, and everyday purposes [3]. Despite their cultural prominence, such ornaments are largely underrepresented in public datasets used in computer vision and artificial intelligence (AI) research [[4], [5]]. As AI-driven applications in fashion, heritage preservation, and e-commerce expand, the need for structured, culturally inclusive datasets has become increasingly evident. The motivation behind compiling this dataset stemmed from the absence of reliable, annotated visual data for traditional Asian ornaments in existing repositories. Most available datasets in this domain are either Western-centric or focused on general accessories without detailed categorization. To address this gap, images were manually collected from various locations within Mirpur, Dhaka, capturing a wide range of ornament styles found in local markets and rural communities. The dataset is designed to serve as a standardized resource for fine-grained classification, object detection, and recognition model development. This data article complements ongoing research into ornament recognition by providing high-quality images suitable for model training, testing, and benchmarking, thereby supporting further innovation in cultural object classification using AI.

## 3 Data Description

**Fig. 1:**
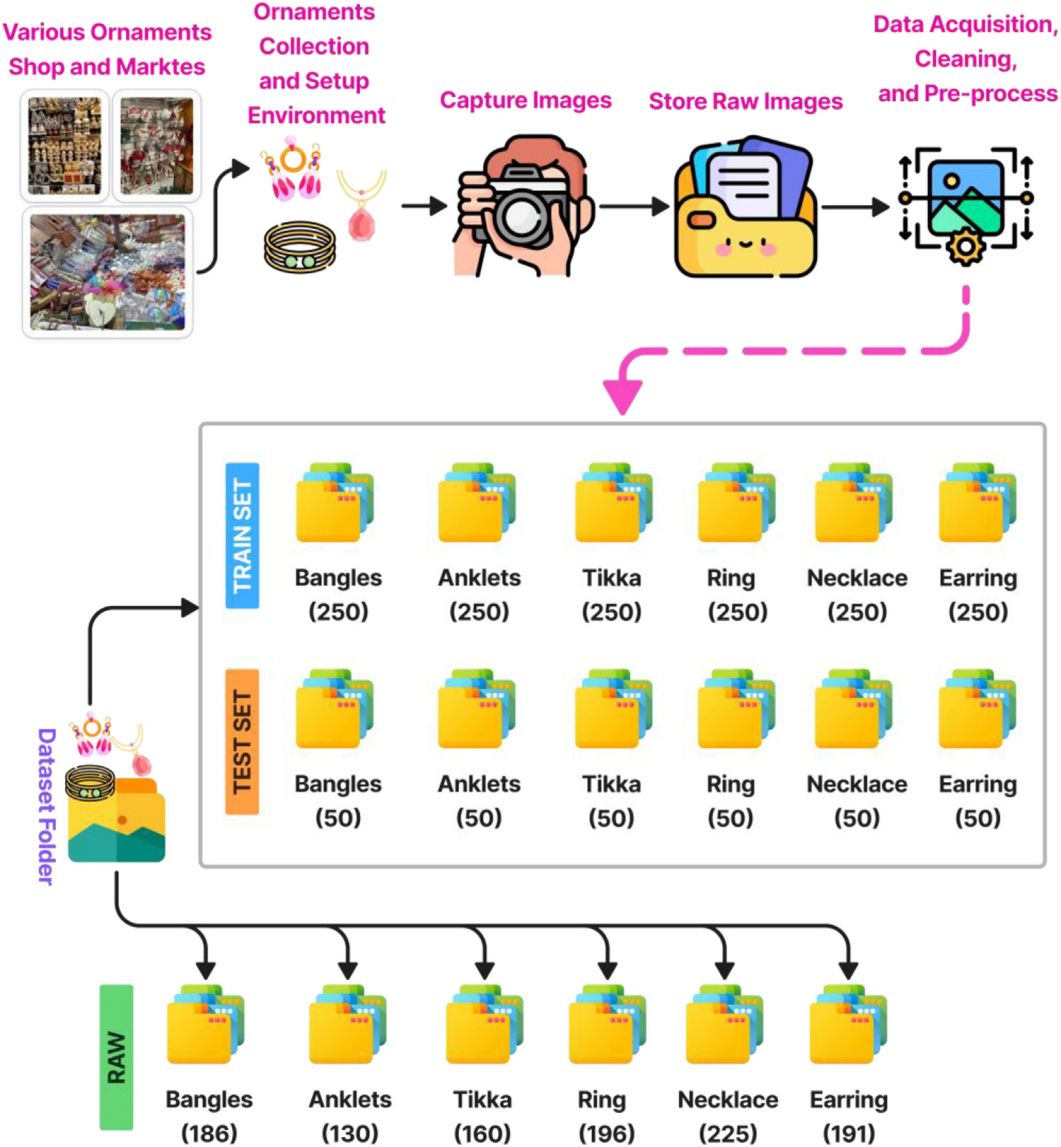
Dataset Collection Procedure and Organization Workflow.

The dataset titled “**OrnAsia**” [6] is organized into labeled folders corresponding to six ornament categories. A total of 1088 high-resolution images is included, stored in JPEG (.jpg) format. The data repository is available at Mendeley Data Repository, and the directory structure is shown in Fig. 1. The figure illustrates the complete workflow used to collect, organize, and prepare the Asian Ornaments ‘OrnAsia’ Dataset for machine learning and computer vision applications. The process begins with sourcing data from various ornament shops and local markets in Mirpur, Dhaka, Bangladesh. These locations were selected to reflect a wide range of traditional ornament designs representative of Bengali and broader South Asian culture. Six ornament categories, Bangles, Anklets (Payel), Tikka, Rings, Necklaces, and Earrings, were selected for inclusion. Ornaments were photographed using smartphone cameras in ambient natural lighting conditions, typically between 25 °C and 28 °C. Images were taken from multiple angles and positions to ensure diversity in visual representation. These images were stored as raw JPEG files and organized according to class. Once captured, the images were reviewed, filtered for quality, and renamed. Blurry or duplicate images were excluded to ensure only clean and representative samples remained. Following this, images were resized, normalized, and labeled appropriately. The final dataset was structured into two version: (i) Raw Dataset version which contains 1088 original images data, and (ii) Computer Vision experiment version which contains a training set with 250 images per class (by augmentation) and a testing set with 50 images per class a total of 1800 images. The raw data folder contains the original, unprocessed images: Bangles (186), Anklets (130), Tikka (160), Ring (196), Necklace (225), and Earring (191). This workflow, illustrated in Fig. 1, ensures a comprehensive, high-quality dataset suitable for AI-driven ornament classification tasks. Table 1 represents a summary of our dataset classes and sample images.

**Table 1:** Dataset Description and Sample Images.

Class Name	Sample Image	Description
**Bangles**		Circular ornaments worn on the wrist, traditionally made from metal or glass, often in sets, showcasing twisted and colourful designs.
**Anklets (Payel)**		Delicate ornaments worn around the ankles, typically made with string and small metal beads or bells, are common in South Asian traditional attire.
**Tikka**		Forehead jewellery, usually attached to the hair and draping down to the centre of the forehead, is often used in bridal and festive looks.
**Ring**		Finger ornament made of metal, often embellished with stones or gems; a common and widely worn accessory across genders.
**Necklace**		Neck adornment crafted from beads, chains, or pendants, typically worn in ceremonies and formal occasions to accentuate traditional wear.
**Earrings**		Ear-worn ornaments, often dangling or stud-style, featuring intricate metalwork, stones, and colourful beads in ethnic designs.

## 4 Experimental Design, Materials, and Methods

The Asian Ornaments Dataset was compiled through a systematic data collection and organization process designed to support computer vision tasks such as object detection, fine-grained classification, and cultural artifact recognition. Images were captured across physical locations in the Mirpur area of Dhaka, Bangladesh, specifically from Mirpur-10 through Mirpur-13. These areas host diverse local markets and ornament shops, offering an authentic representation of traditional South Asian jewelry items. Six classes of ornaments were selected for inclusion: Bangles, Anklets (Payel), Tikka, Rings, Necklaces, and Earrings. The selection criteria focused on cultural relevance, visual distinctiveness, and representation in day-to-day or ceremonial use across Bengali communities. Items were collected and photographed at source under natural lighting to maintain their original color, shine, and texture. All items were captured either on clean white surfaces or with controlled light backgrounds to minimize background noise and maximize object clarity. Images were taken using two smartphone models:

i. Realme Narzo 20 Pro (48 MP, f/1.8 sensor)
ii. Realme Narzo 50 (50 MP AI Camera, f/1.8 aperture)

Smartphones were used for mobility and access to narrow market areas. Flash was turned off, and default camera settings were used without third-party enhancements. The average temperature during capture ranged between 25 °C and 28 28 °C. Multiple images of each ornament were taken from different angles to capture depth, form, texture, and natural variations. Blurred or overexposed samples were discarded immediately. Each image was reviewed and labeled manually by three independent annotators with domain familiarity. Annotation consistency was ensured by applying majority voting in ambiguous cases and conducting two rounds of cross-validation. The dataset includes 1088 labeled images in JPEG format, divided into class-specific folders. Image resolutions varied, but most were between 1800 × 4000 and 2300 × 4600 pixels. Following acquisition, a manual filtering process was applied to remove duplicates, out-of-focus images, and samples with poor lighting or obstructed views. The remaining high-quality images were then resized to a consistent resolution of 1800 × 1800 pixels for uniformity and ease of processing in deep learning frameworks. After labeling, images were organized into training and test sets. All images were resized to 256 × 256 pixels to ensure input uniformity for neural networks. Images retained original color space (RGB) and file format (.jpg). The dataset structure and data capturing process are illustrated in Fig. 1. All data preprocessing was performed using ACDSee Ultimate Photo Studio Pro [7] image processing software. Class balance was preserved across splits, and duplicate checks were conducted using hash values to ensure sample uniqueness. The final dataset is publicly hosted on Mendeley Data under the CC BY 4.0 license, enabling reuse, modification, and integration into machine learning workflows.

## Limitations

Not applicable.

## Ethics Statement

The current work does not involve human subjects, animal experiments, or any data collected from social media platforms.

## Credit Author Statement

**Md. Darun Nayeem:** Investigation, Software, Validation, Formal Analysis, Methodology, Resources, Data Curation, Writing - Original Draft, Visualization. **Saima Zannat Sraboni:** Data Curation. **Shejuti Shithi Biswas:** Data Curation. **Md. Masudul Islam:** Project administration, Conceptualization, Methodology, Formal analysis, Resources, Writing - Original Draft, Visualization.

## Data Availability

Mendeley DataOrnAsia: Asian Ornament Image Dataset (Original data).
